# Genomic-based microsatellite development for *Ternstroemia* (Pentaphylacaceae) and transferability to other Ericales

**DOI:** 10.1007/s11033-023-08258-y

**Published:** 2023-02-14

**Authors:** Hernán Alvarado-Sizzo, Othón Alcántara-Ayala, David Espinosa, Gerardo Rivas, Ken Oyama, Isolda Luna-Vega

**Affiliations:** 1 Laboratorio de Biogeografía y Sistemática, Departamento de Biología Evolutiva, Facultad de Ciencias, Universidad Nacional Autónoma de México, Ciudad Universitaria, Circuito Exterior, 04510 Mexico City, Mexico; 2 Facultad de Estudios Superiores Zaragoza, Universidad Nacional Autónoma de México, Batalla del 5 de Mayo, Ejército de Oriente, 09230 Iztapalapa, Mexico City, Mexico; 3 Departamento de Biología Comparada, Facultad de Ciencias, Universidad Nacional Autónoma de México, Ciudad Universitaria, Circuito Exterior, 04510 Mexico City, Mexico; 4 Escuela Nacional de Estudios Superiores (ENES) Unidad Morelia, Universidad Nacional Autónoma de México, Ant. Carretera a Pátzcuaro 8701. Ex Hda. de San José del Cerrito, Morelia, Michoacán Mexico

**Keywords:** Cloud forest, Genotyping, High throughput sequencing, Illumina MiSeq, Population genetics, SSRs

## Abstract

**Background:**

The genus *Ternstroemia* is associated with the vulnerable tropical montane cloud forest in Mexico and with other relevant vegetation types worldwide. It contains threatened and pharmacologically important species and has taxonomic issues regarding its species limits. This study describes 38 microsatellite markers generated using a genomic-based approach.

**Methods and results:**

We tested 23 of these markers in a natural population of *Ternstroemia lineata*. These markers are highly polymorphic (all loci polymorphic with 3–14 alleles per locus and expected heterozygosity between 0.202 and 0.908), most of them (19 out of 23) are in Hardy-Weinberg Equilibrium and free of null alleles (18 out of 23). Also we found no evidence of linkage among them. Finally, we tested the transferability to six other American species of *Ternstroemia*, two other Pentaphylacaceae species, and four species from different families within the order Ericales.

**Conclusions:**

These molecular resources are promising tools to investigate genetic diversity loss and as barcodes for ethnopharmacological applications and species delimitation in the family Pentaphylacaceae and some Ericales, among other applications.

## Introduction

The genus *Ternstroemia* Mutis ex L.f. contains between 110 and 160 species mainly distributed across the tropical and subtropical regions worldwide [1, 2]. In Mexico, some *Ternstroemia* species are considered either diagnostic or associated with tropical montane cloud forests (TMCF) [3], while other species across the world are widespread in both tropical and temperate forests. According to climate-change scenarios, the TMCF will face severe threats regarding physiological adaptation and survival [4]. Among the species of *Ternstroemia*, some taxa such as *T. dentisepala* B.M. Barthol. and *T. huasteca* B.M. Barthol. are considered a priority for conservation since they are endemic and geographically rare [5]. *Ternstroemia huasteca* is considered “vulnerable” in the IUCN Red List [6]. Moreover, Mexican species such as *T. chalichophila*, *T. dentisepala*, and *T. impressa* belong to the *Ternstroemia lineata* species complex, a taxonomic group with unresolved relationships, putatively obscured by ongoing interspecific processes [7].

In Mexico, most genetic diversity research on forest species has been focused on timber species, such as the Pinaceae and the genus *Quercus*. In contrast, the knowledge of genetic patterns in other tree species is relatively scarce [8]. Nevertheless, some studies on Mexican TMCF species such as *Abies* [9], *Chiranthodendron* [10], *Liquidambar* [11], and *Podocarpus* [12] have identified historical gene drift and populational isolation during the Pleistocene interglacial periods [13].

The biogeographic history of *Ternstroemia* remains poorly known because there are no comprehensible explanations of its amphipacific distribution. However, efforts like the study of Rose et al. [14] included few Pentaphylacaceae species. Moreover, there are no populational studies of any species of the genus nor specific genetic markers, while during recent years, new species are increasingly being described, frequently endemic and/or threatened [15–18]. In Mexico, several species of *Ternstroemia* are considered an ethnopharmacological resource, named as “té de tila”. The dry fruits are used to treat anxiety and insomnia; also, they are known for their analgesic, anti-inflammatory, and anticonvulsant properties [19]. Although, most of the effects result from the neurotoxicity induced by terpenoids and may be considered a health risk [20]. This issue is a health concern because infusions are sold as mixtures with other species (by local sellers and street markets) or in sachets (finely ground in supermarkets).

Currently, there are no published assessments about the impact of the exploitation of *Ternstroemia* fruits on its genetic diversity and demography. However, similar systems (trees under some exploitation) such as *Aquilaria* [21], *Cedrus* [22], and *Dipterocarpus* [23] have been genetically evaluated using microsatellites or simple sequence repeats (SSRs). This technique offers advantages such as codominance, high polymorphism, individual resolution, technical simplicity, and the ease of applying it to degraded DNA, such as herbarium vouchers [24].

Considering the pharmacological importance and ubiquity of *Ternstroemia* within the Mexican TMCF, it is urgent to develop specific genetic markers for the genus. Therefore, this manuscript describes the development of a set of 38 nuclear microsatellites. Specifically, we seek to test its utility in (1) estimating genetic diversity in *Ternstroemia lineata*, and (2) its transferability to six other key species of *Ternstroemia* and other Ericales.

## Materials and methods

### Tissue collection and DNA isolation

**Fig. 1:**
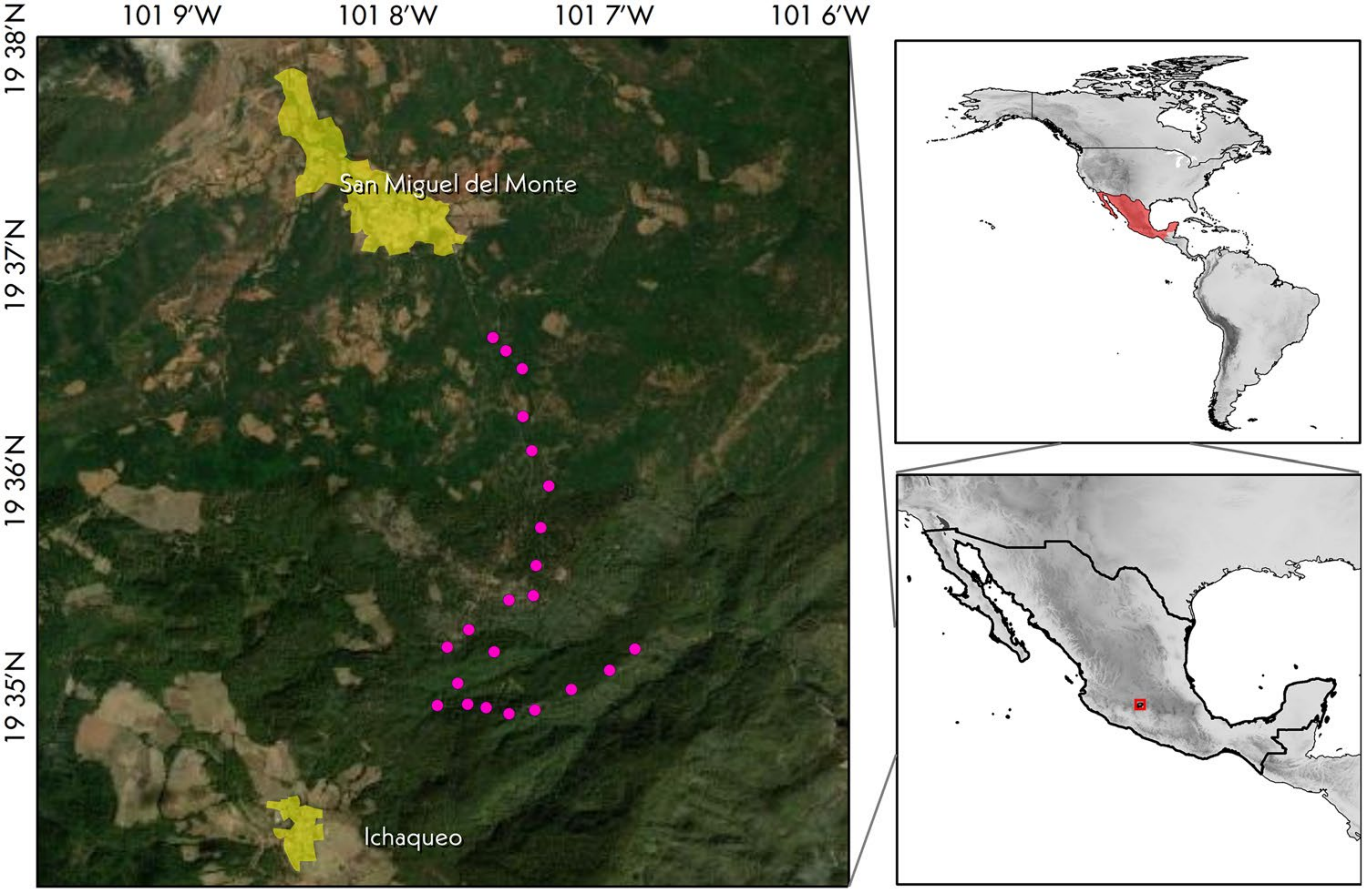
Location of the *Ternstroemia lineata* population sampled for microsatellite evaluation. Each pink dot represents individual coordinates

We collected single, young leaves in silica gel from 20 individuals of *Ternstroemia lineata* from a mixed temperate forest in southern Morelia (Central-Western Mexico), between the localities of San Miguel del Monte and Ichaqueo. The individuals were georeferenced and sampled at least 250 m apart (Fig. 1). Leaves from species other than *T. lineata* were either opportunistically collected and dried in silica gel or retrieved from herbarium vouchers (such as *Freziera* sp. and the South American *T*. *asymmetrica* and *T*. *subserrata*). These two South American species may give insights about the markers’ transferability success in non-Mexican species. The Ericales sample included some representative families across the order: Ebenaceae (*Diospyros xolocotzii*), Foquieriaceae (*Foquieria splendens*), Primulaceae (*Rapanea* sp.), and Symplocaceae (*Symplocos citrea*); this selection is intended to provide a rough estimation of cross-amplification success across the order Ericales. We grounded the leaf tissue of all the studied species in individual 2 mL microtubes in a Retsch Mill (MM400). DNA was isolated using the CTAB protocol [25].

### Microsatellite search using high throughput sequencing

In order to find the repetitive regions, we outsourced AllGenetics & Biology SL (La Coruña, Spain). One individual was randomly chosen as the source sample and used to construct the genomic DNA library. The library was prepared using the Nextera XT DNA enrichment kit (Illumina) with the microsatellite motifs: AC, AG, AT, ACG, and ATCT and following the manufacturer’s instructions. The library was then sequenced in the Illumina MiSeq platform (PE300), producing 9,921,869 paired-end reads. The quality of the raw sequencing data was checked using FastQC [26]. Finally, reads were processed in Geneious 10.2.3 and using in-house developed scripts. Primer design was implemented in Primer3 [27, 28] in Geneious 10.2.3 (Biomatters, LTD).

### Validation and cross-amplification

First, we filtered the database to select only perfect and uninterrupted motifs, and then we randomly selected 38 candidate primer pairs. These 38 loci contained only dinucleotide repeats; by far, the most common (35 loci or 92.1%) motif was (AG)_n_. Two markers, Tli161 and Tli198, presented the motif (AC)_n_. The motif (AT)_n_ was present only in the locus Tli175. PCRs were performed using the Platinum Master Mix (Thermo-Fisher, USA) following the manufacturer’s instructions for reaction assembly and program. The annealing temperature was 60 °C since all the primers were designed around this value. Once the 38 markers were validated in the source *T. lineata* subsp. *lineata* sample, they were tested in six American *Ternstroemia* spp., *Cleyera* Thunb., and *Freziera* Willd. (Pentaphylacaceae), and other Ericales such as *Diospyros* L. (Ebenaceae), *Fouquieria* Kunth. (Fouquieriaceae), *Rapanea* Aubl. (Primulaceae), and *Symplocos* Jacq. (Symplocaceae). Out of the 38 candidate markers, we chose 23 “well-working” primer pairs that showed consistent amplification in a 2% agarose gel electrophoresis (high transferability and amplification success across the genus *Ternstroemia*, few secondary bands and/or bands consistent with the size obtained in the source sample validation). Since the remaining 15 markers show variable amplification success, we decided not to use them in genotyping. However, we report the primer sequences and the expected size. The 23 “well-working” markers subset was fluorescently labeled in the 5′ endings using the Dye Set 33 (Applied Biosystems, USA) and then used to evaluate the parameters of the source sample population. Fragment analysis was performed in Psomagen Inc. (Maryland, USA), and genotyping was achieved using the Microsatellite plugin (v. 1.4.7) of Geneious Prime 2022 (Dotmatics, NZ). Allele scoring was performed manually using as guidelines the supplementary material of Selkoe and Toonen [24]. Genetic parameters such as expected and observed heterozygosity (*He*, *Ho*), allelic richness (Na), and deviation from Hardy-Weinberg equilibrium were calculated in GenAlEx 6.503 [29]. PIC was calculated using PIC_CALC [30]. We tested linkage disequilibrium among all the 23 chosen loci using the association index ($\bar{r}_{d}$) of Agapow and Burt [31] implemented in the R package *poppr* [32]. We checked the presence of null alleles using the R package *PopGenReport* [33]. Finally, we performed an interpolation of the individual heterozygosity using Empirical Bayesian kriging [34] in ArcGIS 10.3 (ESRI, USA), to explore the marker set’s potential in fine-scale genetic approaches. This approach automatically calculated the semivariogram from 1000 simulations using a standard circular neighborhood search (10–15 neighbors data points, radius = 0.013).

## Results and discussion

**Fig. 2:**
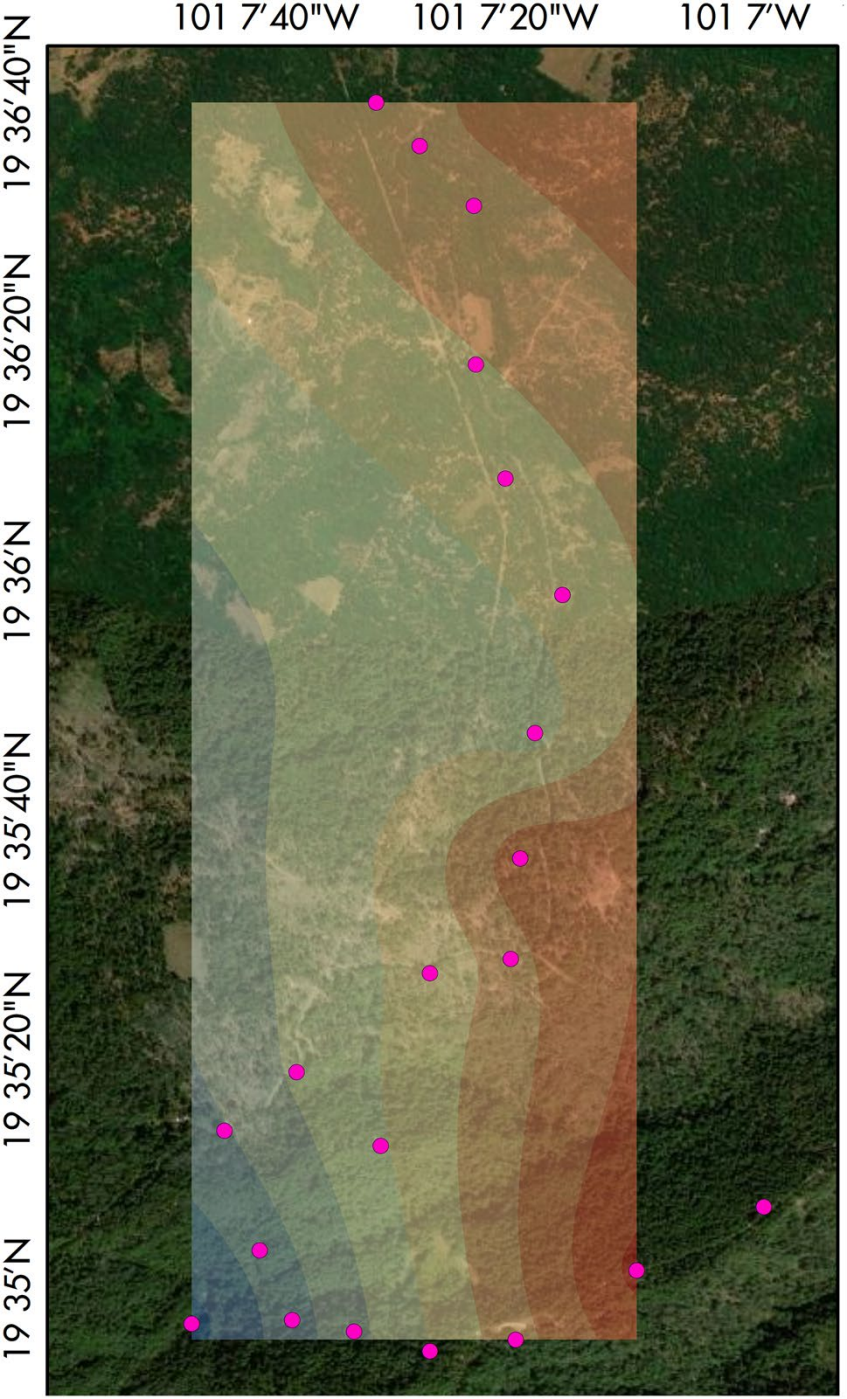
Empirical Bayesian kriging of individual heterozygosity. Satellite image from Google Satellite

All the evaluated markers presented polymorphism. The number of alleles (*Na*) ranged from 3 to 14 (mean = 7.174). The observed heterozygosity ranged from 0 to 0.889 (mean = 0.539), and the expected heterozygosity (*He*) ranged from 0.202 to 0.908 (mean = 0.676). Polymorphism Information Content ranged from 0.19 to 0.90 (mean = 0.643), with only four loci under PIC = 0.5 (Table 2). We found significant evidence of null alleles in five loci, whereas four loci exhibited Hardy-Weinberg disequilibrium (at P > 0.05) (Table 2). The index of association ($\bar{r}_{d}$) was 0.0301 (P = 0.618), indicating no evidence of linkage disequilibrium among the markers. All amplicons, including the microsatellite regions, were deposited in NCBI Genbank (accessions in Table 1). For the candidate loci, transferability is relatively high among *Ternstroemia* spp. (81.6–100%) and the two Pentaphylacaceae (89.5%for *Cleyera theaeoides* (Sw.) Choisy and 81.6%for *Freziera* sp.) (Table 3). Across the Ericales, the amplification success (Table 4) was very low in *Diospyros xolocotzii* Madrigal & Rzed. (7.9% to low in *Symplocos* sp. (26.3%) but consistent with the expected amplification success among families of the same order [35]. Since we selected the 23 evaluated markers for their consistent amplification, the minimum amplification success of this subset for Pentaphylacaceae was 91.3%. The interpolated map of genetic diversity (measured as individual heterozygosity) showed a clear declining trend from South-West (Fig. 2). Therefore, these markers are a suitable set for fine-scale population genetic research in this family. They can also clarify the taxonomic limits among troublesome groups such as the *Ternstroemia lineata* species complex (e.g., *T. chalicophila* Loes., *T. dentisepala*, and *T. impressa* Lundell tested in this study). In this species complex, efforts using traditional phylogenetic markers have been insufficient. They are also useful for assessing the genetic diversity in threatened species, as is the case of *T. huasteca*.

**Table 1:** The 38 candidate loci and the selected 23 (in boldtype) for populational evaluation

Locus	Forward primer (5′–3′)	Reverse primer (5′–3′)	Allelic range or expected size	Motif	Genbank accession
**Tli008**	CAGGGTCAAGTTCCGTTTGT	TTGTTTCCATCTCCCAGGAC	221–227	AG	OP292249
Tli010	TACAAGGTGGGAAGACCAGG	TTCATGGTAACCCTTCCAGC	111	AG	OP292250
Tli013	ACCAATCAATCCAAATCCCA	CTCCAAACGCAAATCCACTT	107	AG	OP292251
Tli024	GCAGACCTCGACAACAATGA	CGCTACTCTGGTCTCCTTGG	107	AG	OP292252
Tli053	CACTTAAACGCAGGCATGAA	GTGGATGGAAAGCGAAGTGT	93	AG	OP292253
**Tli057**	ACGTACATGAAATCTGGCCC	TGGCACATTACCCATCTTGA	87–95	AG	OP292254
**Tli070**	GTCTTCACCTCACCTGCACA	GAAATAGGCCATGAAAGCGA	106–122	AG	OP292255
**Tli072**	AGATTGGGTCAATGGTGCAT	ATAATTGCGTTGTCGGCTTC	99–119	AG	OP292256
**Tli085**	CCTCCCTGTTTCTAGGGTCC	TCGAAACAGCCCAAGTAGGT	81–99	AG	OP292257
**Tli086**	GCTGAGAAGAAATGGCCTTG	TTAAATGCAACGAATGCAGG	140–166	AG	OP292258
**Tli088**	TATGACCATGCTCCACTCCA	GCACAAGGGACACAGAAACA	131–169	AG	OP292259
Tli091	TACTGTGCATGTGCCATTGA	GTGAGGAGGGAGAGGGTTTC	147	AG	OP292260
**Tli093**	TATGAAGGTCCCACCAGACC	GGTTGATCATTCAGGATGGG	92–126	AG	OP292261
**Tli096**	GTGGAGTTGAATGGGTCGTT	CCGATTCTTTGCTCTTCACC	89–111	AG	OP292262
**Tli106**	CACAGACCTCCACAGCCATA	ATCACCATGCCACATCTTCA	100–126	AG	OP292263
**Tli107**	TGGTGATGCTACAGACAGGG	ATGATAGCCAATCCCACTCG	177–187	AG	OP292264
Tli114	AGGAGGGCCATTTCTTCAGT	CCTTCCTCTTTCTCCACCCT	115	AG	OP292265
**Tli118**	ACTTCATGCTTTGAGCAGCA	ACAGGGAAAGAGCAAGGACA	105–123	AG	OP292266
Tli119	CAAACTCCGATCCACAAACC	CGGAGATTTCCGACTGAGAG	100	AG	OP292267
**Tli123**	CTCCCATTTCCATGCACTTT	AACAATGTCTCGGCCATCAT	95–109	AG	OP292268
Tli130	TCAAACTGCACAGCCATGTT	AGTGATCATTGTCACCGCAA	116	AG	OP292269
**Tli149**	TCCGCTGAGGTAGGTGAGAT	GGATCATCAAGGTGCCAATC	184–206	AG	OP292270
Tli161	TCAGCGGCACGTACATTATC	TTCATGACTTTCCGATGCAA	103	AC	OP292271
**Tli162**	ATGATGAGGATACGCTTGCC	CATAGCTAGGTTTCGGTGGG	134–146	AG	OP292272
Tli173	AACTCGTGCTCCCACTTCAC	TCTGCCTTGTTCTGGAGGAT	192	AG	OP292273
Tli174	TAAGTGGGTTGGCCACAAAT	GTACAGTGGGAGGCCTCTTG	118	AG	OP292274
**Tli175**	GGATCTCCTTCATCGCTGTC	AACTCAACCAAACCCACCAC	99–147	AT	OP292275
Tli181	ACAGGCACCACACTTGTGAC	CCAACTTTCGACATCAAGCA	99	AG	OP292276
**Tli182**	TACTGAATTGGTGCTCGGTG	TGGGCTCCTCCTGTAAAGTG	157–183	AG	OP292277
**Tli186**	GACCAACTCAGCCTAAGCCA	GCTTCAATTTACGCCTTTGC	89–109	AG	OP292278
**Tli187**	GCAGTGCAAAGAGCTGACAA	TGACAAATCCACCCAAACAA	86–102	AG	OP292279
**Tli196**	AACGACTTCTCAACCAACCG	GAGTGACAGCCAAGCGAAAT	76–106	AG	OP292280
Tli197	AATGGGTTCTTCACGCTTGT	AAGGAAAGGATATGGCCACC	131	AG	OP292281
**Tli198**	AACCTTCCAATTCAACTGCG	AGAAACATGAAATCCGCCAA	141–195	AC	OP292282
**Tli200**	CTCCTTCATTCCCAGTGGTC	TGATCCCAACCAGAACAACA	85–96	AG	OP292283
Tli205	GGGCCAGTGCATTAAATGAT	CTTGGTGTGCCTGTGTTTGT	110	AG	OP292284
**Tli206**	GAAGCTTTCCAGCCTTCTCC	TCTTCGGTCGACCAGTTACC	63–89	AG	OP292285
Tli208	AGGAAAGGGTCATTTCAGGC	CCTTATTGCAAATGTGCGTG	96	AG	OP292286

**Table 2:** Genetic parameters of the 23 evaluated loci

Locus	Na	*Ho*	*He*	PIC	HWD	Null Alleles
Tli008	4	0.583	0.517	0.482	NS	S
Tli057	5	0.611	0.543	0.503	NS	S
Tli070	3	0.188	0.568	0.482	NS	NS
Tli072	7	0.526	0.717	0.67	NS	NS
Tli085	9	0.571	0.844	0.826	NS	NS
Tli086	7	0.667	0.719	0.686	NS	NS
Tli088	14	0.889	0.841	0.826	NS	S
Tli093	7	0.813	0.658	0.609	NS	S
Tli096	7	0.684	0.668	0.635	NS	NS
Tli106	9	0.722	0.846	0.828	NS	NS
Tli107	3	0.111	0.202	0.19	NS	NS
Tli118	5	0.5	0.642	0.603	S	NS
Tli123	5	0.526	0.575	0.53	S	NS
Tli149	8	0.588	0.732	0.693	NS	NS
Tli162	3	0.263	0.234	0.215	NS	S
Tli175	13	0.556	0.878	0.867	NS	NS
Tli182	14	0.588	0.908	0.901	S	NS
Tli186	8	0.579	0.803	0.78	NS	NS
Tli187	7	0.778	0.796	0.77	NS	NS
Tli196	12	0.833	0.895	0.886	NS	NS
Tli198	3	0	0.531	0.468	NS	NS
Tli200	4	0.294	0.666	0.604	S	NS
Tli206	8	0.526	0.766	0.744	NS	NS

*Na*  allelic richness, *Ho* observed heterozigosity, *He* expected heterozigosity average, *PIC* polymorphism information content, *HWD*  significance of Hardy-Weinberg Disequilibrium at P > 0.05; *S* significant, *NS* no significant, *Null Alleles* evidence of Null alleles, *S* significant, *NS* no significant

**Table 3:** Transferability across Pentaphylacaceae, including *Ternstroemia* spp

Locus	*T. asymmetrica*	*T. chalichophila*	*T. dentisepala*	*T. huasteca*	*T. impressa*	*T. subserrata*	*Cleyera theaeoides*	*Freziera * sp.
**Tli008**	+	+	+	+	+	++	+	+
Tli010	+	+	?	+	+	+	+	+
Tli013	+	+	-	+	+	+	+	-
Tli024	+	++	+	+	+	+	+	+
Tli053	++	+	+	+	+	++	+	?
**Tli057**	+	+	+	+	+	+	+	+
**Tli070**	+	+	+	+	+	+	+	+
**Tli072**	++	+	+	+	+	++	?	+
**Tli085**	+	+	+	+	+	+	++	+
**Tli086**	+	+	+	+	+	+	+	+
**Tli088**	+	+	+	+	+	+	+	+
Tli091	++	+	+	+	+	++	++	+
**Tli093**	+	+	+	+	+	+	+	+
**Tli096**	+	+	+	+	+	+	+	+
**Tli106**	+	+	+	?	+	+	++	?
**Tli107**	+	+	+	+	+	+	+	+
Tli114	+	+	+	+	+	+	+	+
**Tli118**	+	+	+	+	+	+	+	+
Tli119	+	+	+	+	+	+	+	–
**Tli123**	+	+	+	+	+	+	+	+
Tli130	+	+	+	+	+	++	+	+
**Tli149**	+	+	+	+	+	++	+	+
Tli161	+	+	+	+	+	+	+	+
**Tli162**	++	+	+	+	+	++	+	+
Tli173	+	+	–	+	+	+	+	?
Tli174	–	+	+	++	+	++	+	+
**Tli175**	+	+	+	+	+	?	+	+
Tli181	+	+	+	+	+	+	+	+
**Tli182**	+	+	+	+	+	?	+	++
**Tli186**	+	+	+	+	+	+	+	+
**Tli187**	+	+	+	+	+	+	+	+
**Tli196**	+	+	+	+	+	+	+	+
Tli197	+	+	+	+	+	+	+	+
**Tli198**	++	?	+	–	+	+	+	+
**Tli200**	+	+	+	+	+	+	+	+
Tli205	+	+	+	–	+	+	+	+
**Tli206**	+	+	+	+	+	+	+	+
Tli208	+	+	?	+	+	+	++	+

(+) = positive amplification, (++) = several bands besides the expected one, (?) = unespecific amplification, ( –) = no amplification. Loci in boldtypes are those evaluated

**Table 4:** Transferability across some Ericales

Locus	*Diospyros xolocotzii*	*Foquieria splendens*	*Rapanea sp.*	*Symplocos citrea*
**Tli008**	–	–	–	–
Tli010	–	–	-	–
Tli013	–	–	++	–
Tli024	–	–	-	–
Tli053	+	+	+	+
**Tli057**	–	–	–	–
**Tli070**	–	+	++	++
**Tli072**	–	–	++	–
**Tli085**	–	–	–	–
**Tli086**	–	–	++	–
**Tli088**	–	–	++	++
Tli091	–	+	++	+
**Tli093**	+	+	–	++
**Tli096**	–	–	++	+
**Tli106**	–	–	++	+
**Tli107**	–	–	–	–
Tli114	–	–	++	–
**Tli118**	–	+	++	++
Tli119	–	–	++	+
**Tli123**	–	–	++	++
Tli130	–	–	++	++
**Tli149**	–	–	–	–
Tli161	–	–	++	–
**Tli162**	+	+	+	+
Tli173	–	–	–	–
Tli174	–	–	++	++
**Tli175**	–	–	++	++
Tli181	–	–	–	–
**Tli182**	–	–	–	–
**Tli186**	–	–	++	++
**Tli187**	–	–	++	–
**Tli196**	–	+	+	+
Tli197	–	+	++	+
**Tli198**	–	–	++	++
**Tli200**	–	–	+	+
Tli205	–	–	–	–
**Tli206**	–	–	+	+
Tli208	–	–	++	–

(+) = positive amplification, (++) = several bands besides the expected one, (?) = unespecific amplification, (–) = no amplification. Loci in boldtypes are those evaluated
